# Trends in response rates and respondent characteristics in five National Maternity Surveys in England during 1995–2018

**DOI:** 10.1186/s13690-020-00427-w

**Published:** 2020-05-25

**Authors:** Siân Harrison, Fiona Alderdice, Jane Henderson, Maggie Redshaw, Maria A. Quigley

**Affiliations:** Policy Research Unit in Maternal Health and Care, National Perinatal Epidemiology Unit, Nuffield Department of Population Health, University of Oxford, Old Road Campus, Old Road, Oxford, UK

**Keywords:** Maternity survey, Survey response rate

## Abstract

**Background:**

The National Perinatal Epidemiology Unit in England has conducted five National Maternity Surveys (NMS) at varying intervals since 1995. This paper aims to describe the changes in NMS response rates over time and to compare the demographic characteristics of respondents to each NMS.

**Methods:**

This paper is based on secondary data analysis of the NMS (cross-sectional postal surveys) from 1995 to 2018. All women aged 16 years and over who gave birth in England (and Wales in 1995) during specified time periods from 1995 to 2018 were eligible to be selected. For each survey, between 3570 and 16,000 women who were 3–6 months postpartum were selected at random by the Office for National Statistics, using birth registrations. Women could participate on paper, by telephone (from 2006) or online (from 2010).

**Results:**

The response rate to the NMS decreased from 67% in 1995 to 29% in 2018. The decline was evident across demographic groups. In all NMS, response rates were higher in women who were older (crude prevalence ratios (PR) for 16–24 years versus 30–34 years = 0.51–0.73 (across surveys)), married (crude PR for sole versus married registrants = 0.41–0.62), born in the UK (crude PR for non UK-born versus UK-born = 0.70–0.84), and living in less deprived areas (crude PR for least versus most deprived = 0.42–0.63). However, the association between each demographic characteristic and response varied across surveys, with the youngest women, women who registered the birth of the baby in their sole name, and women living in the most deprived areas becoming relatively less likely to respond over time. In multivariable analysis in 2014 and 2018, the effects of age, marital status, country of birth and level of area deprivation on response were attenuated but all four demographic characteristics remained statistically significantly associated with response.

**Conclusions:**

Response rates to the NMS have declined significantly during the last 23 years. The demographic characteristics associated with response were consistent across surveys, but the size of the effect varied significantly, with underrepresented groups becoming relatively less likely to participate over time. It is important to find strategies to increase response rates, particularly amongst underrepresented groups, and to validate the data collected.

## Background

Surveys are an established tool for collecting population-based health data which are not routinely available from other sources. Population-based health surveys allow us to: document the health and wellbeing of the population and to map changes in health over time; to identify health care needs and areas of inequality; to evaluate the performance of healthcare providers and to establish where reform is necessary to improve patient outcomes. Examples of large health surveys in the UK include: the Health Survey for England (HSE) commissioned by NHS Digital to monitor trends in the nation’s health [1]; the GP Patient Survey funded by NHS England to collect data on the experiences, attitudes and characteristics of patients registered with a GP practice in England [2]; and the Care Quality Commission (CQC) Surveys which assess people’s views of the NHS services that they access [3].

There are various methods for conducting surveys. Traditionally the postal or telephone systems have been used as the methods of choice while more recently there has been a surge in internet-based surveys. Regardless of the specific approach, surveys have the potential to recruit large diverse cross-sections of the population and offer respondent anonymity, rapidity of data collection and cost-effectiveness [4]. However, response rates to internet-based surveys tend to be low [5] and response rates to postal and telephone surveys are falling, and so there is the risk of obtaining samples unrepresentative of their target population. This may affect external validity and introduce bias in the estimates based on the data collected.

Over recent decades, there has been a persistent decline in response rates to surveys and this decline is exemplified in postal surveys into maternal and infant health. The response rates to the Infant Feeding Surveys (IFS) [6–11], the CQC Maternity Surveys [12–17], and the National Maternity Surveys (NMS) [18–20], which are three large surveys of maternal and infant health carried out at regular intervals within the UK, have fallen with each successive survey. The response rate to the IFS declined from 91% in 1985 [6] to 51% in 2010 [11] and the response rates to more recent CQC surveys and NMS have fallen even lower [17, 20]. Such a significant decline brings into question the viability of collecting research data through postal surveys in the future. However, it is not solely the response rate that determines the validity of survey data; the extent to which the response is representative of the target population is key, regardless of the rate of response. Therefore, it is important to establish whether the demographic characteristics of survey respondents are changing as response rates decline, specifically whether surveys are becoming decreasingly representative of their target populations.

The objectives of this paper are to describe the changes in response rates over time to the NMS conducted by the National Perinatal Epidemiology Unit (NPEU) and to compare the demographic characteristics of respondents to each NMS.

## Methods

### Design and participants

This study analysed secondary data from all five completed NMS in 1995, 2006, 2010, 2014 and 2018. The NMS were large population-based cross-sectional postal surveys of women’s health and maternity care during pregnancy and the postpartum period. The samples of women selected for the surveys were identified by the Office for National Statistics (ONS) using birth registration records. The number of women sampled for the surveys increased over time to ensure sufficient data to address the objectives of the surveys (e.g. the sample size for the 2018 survey was calculated to estimate the prevalence of most outcomes with reasonable precision, based on the response rate to a pilot survey). Therefore, random samples of between 3570 and 16,000 women aged 16 years and over who had their babies during specified one- or two-week time periods in England (and Wales in 1995) were selected. Women were 3–4 months postpartum at the time of recruitment from 1995 to 2014, and in 2018, women were recruited later (6 months postpartum) due to the inclusion of additional questions relating to the postpartum period. In the week prior to all mailings for each survey, checks on infant deaths were made by ONS and any women whose babies had died were excluded.

### Procedure

In all NMS, the questionnaires were mailed to women by ONS and returned directly to the research team at the NPEU. Reminder letters and additional questionnaires (from 2006 onwards) were mailed to non-respondents using a tailored reminder system [21]. The surveys were administered via first class Royal Mail and return postage was paid for all surveys. Women were able to complete the questionnaire on paper or, from 2006 onwards, by telephone (with an interpreter if required) or, from 2010 onwards, there was also the option to participate online. For the first time in the 2018 survey, an incentive was offered for participation in the survey in the form of a prize draw for a £500 gift voucher. This was included due to the low response rate to a pilot survey conducted prior to the 2018 survey.

### Measures

The NMS questionnaires all followed a similar format, taking women through their pregnancy, labour and birth, and postnatal care. Topic areas included satisfaction with care, infant feeding, maternal and infant health, and maternal mental health. The questionnaires included predominantly structured questions with multiple-choice items and Likert scales for responses. The questionnaires also included some open questions allowing respondents to provide clarification on specific points and to express their views and describe their experiences in their own words if they wished. Each NMS questionnaire and the accompanying documentation (i.e. recruitment letters, information sheets) built on the previous instrument/documents with some additions and minor adjustments to ensure that current issues of interest were captured. The questionnaires varied between 16 and 44 pages in length.

In summary, the rationale for each of the surveys was the same – to explore women’s health and experiences of maternity care during pregnancy and the postpartum period. The overall design, the sampling frame, the study procedures and the format of the questionnaire were also largely consistent across all NMS from 1995 to 2018. The sample size increased due to decreasing response rates and some other specific aspects of the surveys evolved over time to capture different perinatal issues and to reflect new technologies, emerging literature on how to improve response rates and feedback from previous surveys. Additional details of the survey characteristics are shown in Table 1.

**Table 1:** Study characteristics for the NMS (1995–2018)

Year of survey	1995	2006	2010	2014	2018
**Sample source**	ONS birth registrations	ONS birth registrations	ONS birth registrations	ONS birth registrations	ONS birth registrations
**Number of women sampled**	3570	4800	10,000	10,002	16,000
**Region**	England and Wales	England	England	England	England
**Period of birth**	June–July 1995	March 2006	October–November 2009	January 2014	October 2018
**Baby age at recruitment**	4 months	3 months	3 months	3 months	6 months
**Time of initial mail out**	November	June	January	April	April
**Modes of response available**	Postal	Postal Telephone (interpretation service)	Postal Telephone (interpretation service) Online	Postal Telephone (interpretation service) Online	Postal Telephone (interpretation service) Online
**Incentive**	None	None	None	None	Prize draw for £500 gift voucher
**Number or reminders**	0	2	3	3	2^a^
**Timing of reminders (from initial mail-out)**	N/A	+ 2 weeks: reminder letter + 4 weeks: reminder questionnaire	+ 2 weeks: reminder letter + 4 weeks: reminder questionnaire + 8 weeks: reminder letter	+ 2 weeks: reminder letter + 4 weeks: reminder questionnaire + 8 weeks: reminder letter	+ 3 weeks: reminder questionnaire + 7 weeks: reminder questionnaire
**Length of questionnaire**	44 pages	28 pages	28 pages	28 pages	16 pages

^a^ The number of reminders in the 2018 NMS was reduced at the request of the Research Ethics Committee

### Statistical analysis

The response rate was calculated by dividing the total number of responses (excluding refusals, duplicate and unusable returns) by the total number of women sampled (excluding packs confirmed as undelivered). Undelivered packs were recorded for all NMS except the 1995 survey. Response rates were compared over time both graphically and statistically using Chi-Square analyses. Annual changes in response rates were calculated by dividing the difference in the response rates between successive surveys by the number of years between surveys.

For each survey, ONS provided anonymised aggregate and/or individual-level data on key demographic variables for all of the women selected to enable comparison of the respondents and non-respondents. These data included age-group (16–19 years, 20–24 years, 25–29 years, 30–34 years, 35–39 years or 40+ years), marital status at birth registration (married, joint registration by both parents living at the same address, joint registration by both parents living at different addresses, or sole registration), country of birth (UK or non-UK) and Index of Multiple Deprivation (IMD) for the mother’s area of residence (grouped into quintiles) [22]. Some age-groups were combined in the analysis due to lower numbers of respondents in the youngest and oldest age-groups (16–24 years, 25–29 years, 30–34 years, or 35+ years).

The demographic characteristics associated with response were estimated for each survey separately using crude prevalence ratios (PR) with 95% confidence intervals (CI). Forest plots were used to compare PR for the youngest women (compared to the 30–34-year-old women), the sole registrants (compared to the married registrants), the women born outside the UK (compared to UK-born women), and the women living in the most deprived areas (compared to the women living in the least deprived areas) across the surveys. A test for statistical heterogeneity was performed to ascertain whether the association between each demographic characteristic and response varied over time. A *p*-value of less than 0.10 suggested the presence of statistically significant heterogeneity.

Multivariable analysis was not possible for the three earlier surveys as the available data on non-respondents was aggregate. However, when individual-level data were available (2014 and 2018 only), multivariable logistic regression was performed, with the resulting adjusted prevalence ratios (APR) compared to the crude PR to assess confounding between demographic variables.

## Results

### Response rates over time

Table 2 shows the response rates to the NMS from 1995 through to 2018. The response rate decreased significantly with each successive survey from 67.4% in 1995 to 29.0% in 2018 (*p* < 0.0001). Undelivered packs were not recorded in 1995; hence the observed response rate is likely to be a slight underestimate of the true rate in this survey (the other surveys have had an undelivered rate of between 1.5 and 3.0%). The annual rate of change across the years that the surveys span was: − 0.4% from 1995 to 2006; − 2.1% from 2006 to 2010; − 1.9% from 2010 to 2014; and − 4.4% from 2014 to 2018.

**Table 2:** Response rates to the NMS (1995–2018)

	Year of survey				
	1995	2006	2010	2014	2018
**Number of women sampled**	3570	4800	10,000	10,002	16,000
**Number of undelivered packs/ineligible women (%)**	N/A^a^	73 (1.5%)	149 (1.5%)	216 (2.2%)	472 (3.0%)
**Number of usable responses**	2406	2960	5333	4571	4509
**Response rate (95% CIs)**	67.4% (65.8, 68.9)	62.6% (61.2, 64.0)	54.1% (53.1, 55.1)	46.7% (45.7, 47.7)	29.0% (28.3, 29.8)
**Change from previous survey**	N/A	−4.8%	−8.5%	−7.4%	−17.7%
**Annual rate of change**	N/A	−0.4%	−2.1%	−1.9%	−4.4%

^a^Undelivered packs were not recorded in 1995

**Fig. 1:**
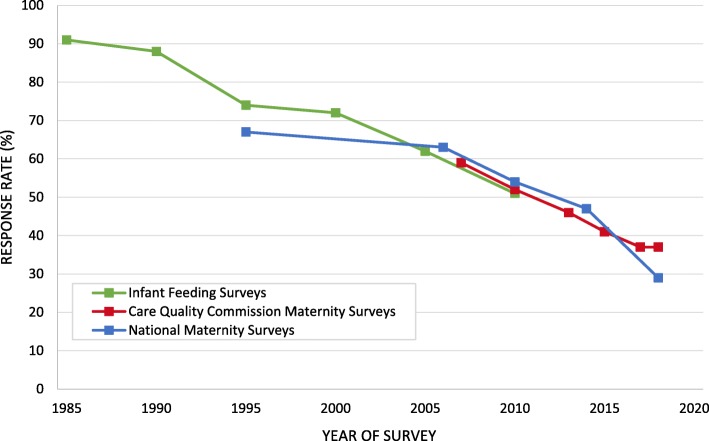
Response rates to the IFS, CQC Maternity Surveys, and NMS (1985–2018)

The declining response rate to the NMS is illustrated in Fig. 1. To place the NMS response rates in context, they are shown alongside the published response rates to the IFS [6–11] and CQC maternity surveys [12–17].

### Demographic characteristics of respondents over time

Table 3 shows the response rates and the PR for the likelihood of response according to demographic characteristics for each survey. A lower response rate to each successive survey is evident across all of the demographic subgroups, with the exception of the mothers born outside of the UK in the 2010 survey, where a marginal increase was achieved compared to 2006.

**Table 3:** Response rates and PR for response rates by demographic characteristics for the NMS (1995–2018)

Year of survey	1995^a^			2006			2010			2014			2018		
Number sampled	***N*** = 3570			***N*** = 4727			***N*** = 9851			***N*** = 9786			***N*** = 15,528		
	%	PR	95%CI	%	PR	95%CI	%	PR	95%CI	%	PR	95%CI	%	PR	95%CI
**Age (years)**															
16–24	51.6	0.66	0.61, 0.70	50.2	0.73	0.68, 0.78	37.5	0.59	0.56, 0.63	31.4	0.58	0.54, 0.63	16.7	0.51	0.46, 0.56
25–29	65.2	0.83	0.79, 0.87	58.9	0.86	0.81, 0.91	51.0	0.81	0.77, 0.84	44.3	0.82	0.78, 0.87	25.3	0.77	0.72, 0.82
30–34	78.7	–	–	68.9	–	–	63.3	–	–	53.8	–	–	32.9	–	–
> = 35	81.5	1.04	0.98, 1.10	68.1	0.99	0.94, 1.05	65.6	1.04	0.99, 1.08	55.0	1.02	0.97, 1.08	36.3	1.10	1.04, 1.17
**Marital status**															
Married	N/A	N/A	N/A	65.9	–	–	60.5	–	–	53.5	–	–	34.9	–	–
Joint registration (same address)	N/A	N/A	N/A	61.3	0.93	0.88, 0.98	52.4	0.87	0.83, 0.90	45.8	0.86	0.82, 0.90	26.7	0.76	0.72, 0.81
Joint registration (different address)	N/A	N/A	N/A	44.2	0.67	0.60, 0.75	34.0	0.56	0.51, 0.62	27.2	0.51	0.46, 0.56	13.2	0.38	0.33, 0.43
Sole registration	N/A	N/A	N/A	40.9	0.62	0.54, 0.72	34.1	0.56	0.50, 0.63	25.8	0.48	0.42, 0.56	14.3	0.41	0.34, 0.49
**Country of birth**															
UK	N/A	N/A	N/A	65.7	–	–	56.8	–	–	48.7	–	–	31.4	–	–
Not UK	N/A	N/A	N/A	45.8	0.70	0.65, 0.75	46.2	0.81	0.78, 0.85	41.1	0.84	0.80, 0.89	23.2	0.74	0.70, 0.79
**Index of multiple deprivation**															
1st (most deprived)	N/A	N/A	N/A	46.4	0.63	0.59, 0.68	40.2	0.58	0.55, 0.62	33.4	0.55	0.52, 0.59	17.2	0.42	0.39, 0.45
2nd	N/A	N/A	N/A	58.2	0.79	0.74, 0.85	48.1	0.70	0.66, 0.73	43.8	0.72	0.68, 0.77	25.5	0.62	0.57, 0.67
3rd	N/A	N/A	N/A	67.8	0.92	0.87, 0.98	59.4	0.86	0.82, 0.90	51.3	0.85	0.80, 0.90	32.2	0.78	0.73, 0.84
4th	N/A	N/A	N/A	70.7	0.96	0.91, 1.03	64.9	0.94	0.89, 0.99	54.9	0.91	0.85, 0.96	37.1	0.90	0.84, 0.97
5th (least deprived)	N/A	N/A	N/A	73.3	–	–	69.1	–	–	60.6	–	–	41.2	–	–

^a^ Data were not available on marital status, country of birth or IMD for non-respondents in the 1995 survey

**Fig. 2:**
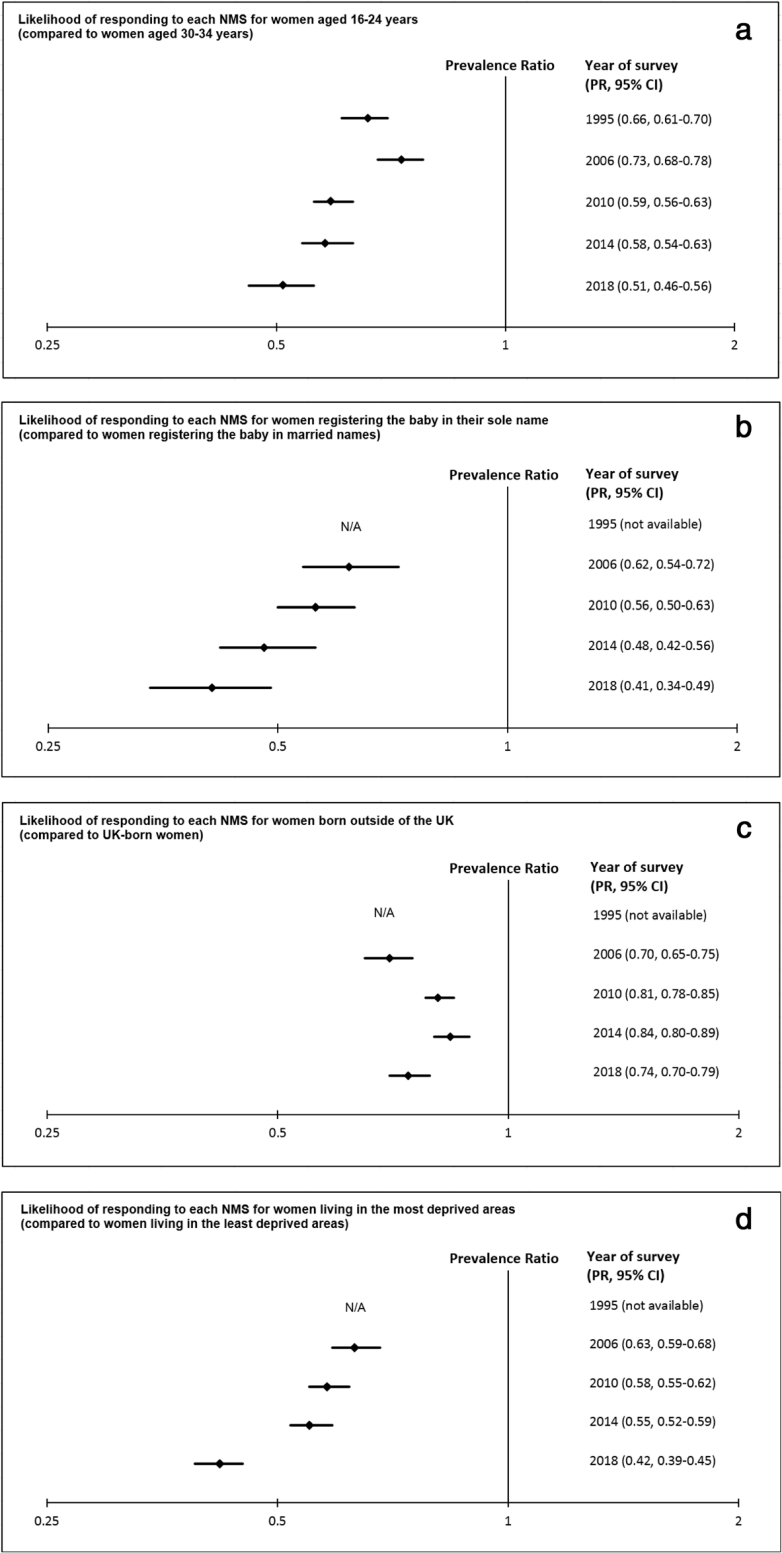
**a**: Likelihood of responding to each NMS for women aged 16–24 years (compared to women aged 30–34 years). **b**: Likelihood of responding to each NMS for women registering the baby in their sole name (compared to women registering the baby in married names). **c**: Likelihood of responding to each NMS for women born outside of the UK (compared to UK-born women). **d**: Likelihood of responding to each NMS for women living in the most deprived areas (compared to women living in the least deprived areas)

Figure 2a-d show the PR for: the youngest women (16–24 years) compared to the 30–34-year-old women; sole registrants compared to married registrants; women born outside of the UK compared to UK-born women; and the women living in the most deprived areas compared to the women living in the least deprived areas.

Women who were in the youngest age category (16–24 years) had a significantly lower response rate to each of the surveys compared to the 30–34-year-old women. The PR associated with being in the youngest age group varied significantly across surveys from 0.51 to 0.73 (Fig. 2a), with a tendency for a larger effect in the more recent surveys (Heterogeneity Chi-Square = 45.68, df = 4, *p* < 0.001). The 25–29-year-old women also had a significantly lower response to each of the surveys compared to the 30–34-year-old women. The response rate for women in the oldest age category (35+ years) was similar to the 30–34-year-old women in the surveys from 1995 to 2014 (PR ranged from 0.99 to 1.04) and slightly higher in the 2018 survey (PR = 1.10) (Table 3).

Women who were unmarried when they registered the birth of their baby had a significantly lower response rate to each of the surveys compared to married women. Table 3 shows that the PR associated with not registering the baby in married names varied across survey years: from 0.76 to 0.93 for joint registrants at the same address; from 0.38 to 0.67 for joint registrants at different addresses; and from 0.41 to 0.62 for sole registrants. The size of the effect for sole registrants, compared to married registrants, varied significantly across surveys (Fig. 2b) and tended to get stronger over time (Heterogeneity Chi-square = 14.83, df = 3, *p* < 0.01).

Women born outside of the UK had a significantly lower response rate to each of the surveys than UK-born women (Table 3). Between 2006 and 2014, women born outside the UK had a lower decline in response rate (45.8 to 41.1%) than UK-born women (65.7 to 48.7%) although the response rates in both groups declined substantially in 2018. The PR associated with being born outside of the UK varied significantly across surveys from 0.70 to 0.84 (Heterogeneity Chi-square = 21.75, df = 3, *p* < 0.001) (Fig. 2c) but there was no clear pattern over time.

The response rate decreased with each drop in quintile of area deprivation in all surveys, for example, ranging from 73.3% (least deprived) to 46.4% (most deprived) in 2006 and from 41.2% (least deprived) to 17.2% (most deprived) in 2018 (Table 3). The PR associated with being in the most deprived quintile, compared to the least deprived quintile, varied significantly across surveys from 0.42 to 0.63 (Fig. 2d), with a larger effect in the most recent survey in 2018 (Heterogeneity Chi-square = 58.06, df = 3, *p* < 0.001).

In summary, the overall pattern of response indicates that women were more likely to respond to each of the surveys if they were older, married at the time of registering the birth of their baby, born in the UK and living in less deprived areas. These demographic characteristics associated with response to the NMS have not altered substantially over this time. However, response to the survey by the youngest women, women who registered the birth of the baby in their sole name, and women living in the most deprived areas has become relatively less likely over time.

Adjusted prevalence ratios (APR) for response based on demographic characteristics were calculated for the 2014 and 2018 surveys (Table 4). The adjusted estimates are very similar across the two surveys. Most of the crude PRs are attenuated after mutual adjustment but women were still significantly more likely to respond if they were older, married, born in the UK and living in less deprived areas, indicating that these demographic characteristics have independent effects on the response rates.

**Table 4:** PR and APR for response rates by demographic characteristics for the NMS 2014 and 2018

Year of survey				2014				2018
Number sampled				***N*** = 9786				N = 15,528
	PR	95%CI	APR	95%CI	PR	95%CI	APR	95%CI
**Age (years)**^a^								
16–24	0.58	0.54, 0.63	0.72	0.65, 0.79	0.51	0.46, 0.56	0.68	0.61, 0.76
25–29	0.82	0.78, 0.87	0.88	0.81, 0.95	0.77	0.72, 0.82	0.86	0.80, 0.93
30–34	1	–	1	–	1	–	1	–
≥ 35	1.02	0.97, 1.08	1.01	0.94, 1.09	1.10	1.04, 1.17	1.11	1.03, 1.19
**Marital status**^a^								
Married	1	–	1	–	1	–	1	–
Joint registration (same address)	0.86	0.82, 0.90	0.91	0.85, 0.98	0.76	0.72, 0.81	0.83	0.77, 0.88
Joint registration (different address)	0.51	0.46, 0.56	0.61	0.54, 0.69	0.38	0.33, 0.43	0.48	0.41, 0.55
Sole registration	0.48	0.42, 0.56	0.57	0.48, 0.68	0.41	0.34, 0.49	0.52	0.42, 0.63
**Country of birth**^a^								
UK	1	–	1	–	1	–	1	–
Not UK	0.84	0.80, 0.89	0.81	0.75, 0.87	0.74	0.70, 0.79	0.72	0.67, 0.77
**Index of multiple deprivation**^a^								
1st (most deprived)	0.55	0.52, 0.59	0.68	0.62, 0.75	0.42	0.39, 0.45	0.56	0.51, 0.62
2nd	0.72	0.68, 0.77	0.83	0.75, 0.91	0.62	0.57, 0.67	0.76	0.69, 0.83
3rd	0.85	0.80, 0.90	0.92	0.84, 1.01	0.78	0.73, 0.84	0.88	0.80, 0.96
4th	0.91	0.85, 0.96	0.95	0.86, 1.04	0.90	0.84, 0.97	0.96	0.87, 1.04
5th (least deprived)	1	–	1	–	1	–	1	–

^a^ Each variable is mutually adjusted in the analysis

## Discussion

There has been a persistent downward trend in the response rate to the NMS from 1995 through to 2018. The characteristics associated with response to the NMS have not altered substantially over this time period due to a decline in the response rate across all demographic groups. Therefore, the decline is not symptomatic of behavioural trends in particular subgroups. Rather, the likelihood of postpartum women choosing to participate in the NMS has greatly reduced over the last 23 years largely irrespective of demographic characteristics. However, it is notable that response by the youngest women, women registering the birth of the baby in their sole name, and women living in the most deprived areas has become relatively less likely over time. If this trend continues, women in these groups will become even more underrepresented in such studies; hence, it is vital to target these women with engagement strategies.

The response rate to each of the five NMS has been consistent with other postal surveys into maternal and infant health conducted within the UK [6–17, 23, 24]. The IFS and CQC Maternity Surveys have taken place over a similar time period to the NMS and response rates across the different surveys have been comparable. For example, surveys carried out consecutively in 2005 (IFS), 2006 (NMS) and 2007 (CQC) returned response rates of 62, 63 and 59% respectively [10, 12, 18]. The three surveys coincided in 2010 and the response rates were all between 51 and 54%. More recently, the NMS and CQC have achieved successive response rates of 46% in 2013 (CQC), 47% in 2014 (NMS), 41% in 2015 (CQC), 37% in 2017 and 2018 (CQC) and 29% in 2018 (NMS). Therefore, it is clear that the decline in response rates over time has occurred across various surveys into maternal and infant health. The decline is also consistent with the trend seen more generally with this method of data collection [25].

According to the literature, there are certain individual characteristics that make participation in research studies more likely. These characteristics include being female, older, married, more educated and having a higher socioeconomic status. This respondent profile has been found across a multitude of studies spanning many years and covering diverse fields of research [25, 26]. Therefore, the fact that the respondents to each of the five NMS have fitted this profile is unsurprising.

Few studies have described trends in survey respondent characteristics over time. The IFS found a significant change in the characteristics of mothers over the longer-term, with the sample becoming older, staying in education for longer, and having higher socio-economic characteristics over time [6–11]. Results from the CQC Maternity Surveys show changes in the age profile of mothers, with age bands representing older women increasing, while age bands for younger women decreasing over time [12–17]. The changes in the characteristics of respondents to the IFS and CQC reflect the changes in characteristics of women giving birth in the UK.

Looking beyond characteristics of individual women, there are several possible methodological explanations for why the response rate to the NMS may have changed over time. Although there was substantial consistency in the methods employed in each of the surveys, there were also variations over the years which may have influenced the response rate. Firstly, the ages of the babies when their mothers were approached differed between surveys, being 6 months in 2018 compared to 3–4 months in previous surveys. This may have adversely affected participation due to the women being further from their experience of pregnancy, labour and birth, and also the increasing demands that may arise later in the postpartum period, such as returning to work. However, in pilot work conducted in preparation for the 2018 survey, response rates for recruitment at 6 months postpartum were less than 3% below response rates for recruitment at 3 months postpartum [27].

Secondly, the available modes of response increased across the surveys, from postal only in 1995 to include telephone and online options in later surveys. Offering greater flexibility in response options might be considered a methodological advancement, yet the concept of “paradox of mode choice” was put forward in a recent meta-analysis of concurrent web options whereby allowing respondents to decide how they complete the survey makes it more likely they won’t complete the survey at all [28].

The length of the questionnaires and the number of reminders also varied across the NMS. In general, the questionnaire became shorter and the number of reminders increased which, according to the literature, should result in higher response rates [29]. Nevertheless, the response rate to the NMS declined despite these developments. One exception to note is that the number of reminders in 2018 reduced from three to two at the request of the ethics committee. Given that number of contacts has been emphasised as one of the most important determinants of response [30], this reversion might partly explain the marked decline in response in the most recent survey. Finally, additional factors such as the time of year at which the surveys were administered and the specific content of the questionnaires and study documentation may have contributed to the changing response rates over time [29, 31].

The trend in the response rates to the NMS is discordant with the existing evidence on how to maximise returns. The response rate to the 1995 survey was high despite employing the longest questionnaire and having no reminder mail-outs and the subsequent surveys have yielded lower returns despite observing recommendations for enhanced methods. This suggests that there are other factors in play, beyond methodological features, which contributed to the declining response to the NMS and which may explain the general and persistent decline in survey response rates.

One possibility is that the proliferation of surveys in circulation imposes an increasing burden on the general public [25, 26]. Linked to the increased demand for participation is the notion of survey fatigue arising from continual requests for individuals to provide feedback on services they have accessed. It may be difficult for survey recipients to distinguish between scientific studies and market research. Furthermore, there may have been an erosion of trust with regard to sharing personal information for the purposes of research, regardless of its source, partly due to publicised data leaks and partly due to a lack of confidence regarding how personal information will be used. This issue may have affected the 2018 survey in particular due to its coincidence with the implementation of the new General Data Protection Regulation in the UK. Further research is required to explore the impact of these factors on research participation. Another possible explanation is that our increased routine use of electronic mail over recent decades could have demoted the value of paper-based mail, hence adversely affecting the reception of postal surveys. However, studies that have employed internet-based surveys have often reported comparable or even lower response rates to those found in other survey modalities suggesting there has been a general decline in participation as opposed to a shift to different response modalities [30, 32, 33].

Given the challenges with recruitment of participants to scientific research, there is the risk that survey returns will continue to diminish. However, it is important to note that response rate alone does not determine the extent of bias and low response rates do not necessarily indicate a high level of bias inherent in a study; the extent to which non-response is associated with the outcome of interest is more important [25]. Therefore, estimates of associations within the data can still be valid even with declining response rates. Furthermore, statistical techniques, such as survey weighting, can be used if potential bias is introduced through non-response [25]. This is not to say that low rates of return should be accepted unreservedly; it is necessary to interpret prevalence rates based on low response rates with caution and it is important to search for innovative strategies to tackle low response rates and to develop novel approaches to data collection. There is a growing literature on methods to increase response rates to surveys [29] and a number of strategies have been shown to be effective.

There are a number of strengths to this analysis. Firstly, the overall design, sampling frame, study procedures and format of the questionnaire were largely consistent across all NMS from 1995 to 2018; this methodological consistency enables comparison of the response rates over a 23-year period. Although the methods were not exactly the same for each NMS, changes introduced in successive surveys were mostly informed by the available evidence for how to optimise returns (e.g. shorter questionnaire, additional reminders); hence they were expected to increase the response rate rather than reduce it. Another strength is that all surveys included random samples of women drawn from a sampling frame of all births in England (and Wales in 1995) during specified time periods. A further important strength is that in contrast to the majority of online surveys, aggregate or individual-level demographic data were available for non-respondents in all NMS, which enabled the assessment of the representativeness of the data.

Aside from the variation in the specific methods employed in each of the NMS, the main limitation to the analysis was the lack of individual-level data on demographic factors for non-respondents for earlier (pre-2014) NMS, which precluded the estimation of adjusted prevalence ratios to identify independent effects. Nevertheless, although a comparison of adjusted and unadjusted estimates for later NMS indicates some confounding between the different demographic characteristics, it does not materially change the observed effects.

## Conclusion

Response rates to the NMS have declined significantly over the last 23 years. The demographic characteristics associated with response were consistent across surveys, but the size of the effect varied significantly, with underrepresented groups becoming relatively less likely to participate over time. The declining response rates to the NMS brings into question the viability of continuing to use the survey method to capture the experiences of postpartum women. However, such data are not routinely available from other sources and currently there is no better alternative method to collect large-scale population-based data. Although estimates of prevalence based on low returns need to be interpreted with caution, measures of effect drawn from such data may still be valid. Nevertheless, it is important to find strategies to halt the decline in survey response rates, particularly amongst underrepresented groups, and to validate the data collected.

## Data Availability

All data generated or analysed during this study are included in this published article and its supplementary information files.

## Abbreviations

APR: Adjusted Prevalence Ratio
CI: Confidence Interval
CQC: Care Quality Commission
IFS: Infant Feeding Survey
IMD: Index of Multiple Deprivation
NHS: National Health Service
NMS: National Maternity Survey
NPEU: National Perinatal Epidemiology Unit
ONS: Office for National Statistics
PR: Prevalence Ratio
